# The LO-BaFL method and ALS microarray expression analysis

**DOI:** 10.1186/1471-2105-13-244

**Published:** 2012-09-24

**Authors:** Cristina Baciu, Kevin J Thompson, Jean-Luc Mougeot, Benjamin R Brooks, Jennifer W Weller

**Affiliations:** 1 Department of Bioinformatics and Genomics, University of North Carolina at Charlotte, Charlotte, NC, 28223, USA; 2 ALS Biomarker Laboratory, Carolinas Neuromuscular/ALS-MDA Center, Department of Neurology, Carolinas Medical Center, Charlotte, NC, 28207, USA; 3 University of North Carolina School of Medicine, Charlotte Campus, Charlotte, NC, 28203, USA

## Abstract

**Background:**

Sporadic Amyotrophic Lateral Sclerosis (sALS) is a devastating, complex disease of unknown etiology. We studied this disease with microarray technology to capture as much biological complexity as possible. The Affymetrix-focused BaFL pipeline takes into account problems with probes that arise from physical and biological properties, so we adapted it to handle the long-oligonucleotide probes on our arrays (hence LO-BaFL). The revised method was tested against a validated array experiment and then used in a meta-analysis of peripheral white blood cells from healthy control samples in two experiments. We predicted differentially expressed (DE) genes in our sALS data, combining the results obtained using the TM4 suite of tools with those from the LO-BaFL method. Those predictions were tested using qRT-PCR assays.

**Results:**

LO-BaFL filtering and DE testing accurately predicted previously validated DE genes in a published experiment on coronary artery disease (CAD). Filtering healthy control data from the sALS and CAD studies with LO-BaFL resulted in highly correlated expression levels across many genes. After bioinformatics analysis, twelve genes from the sALS DE gene list were selected for independent testing using qRT-PCR assays. High-quality RNA from six healthy Control and six sALS samples yielded the predicted differential expression for 7 genes: *TARDBP*, *SKIV2L2*, *C12orf35*, *DYNLT1*, *ACTG1*, *B2M*, and *ILKAP*. Four of the seven have been previously described in sALS studies, while *ACTG1*, *B2M* and *ILKAP* appear in the context of this disease for the first time. Supplementary material can be accessed at: http://webpages.uncc.edu/~cbaciu/LO-BaFL/supplementary_data.html.

**Conclusion:**

LO-BaFL predicts DE results that are broadly similar to those of other methods. The small healthy control cohort in the sALS study is a reasonable foundation for predicting DE genes. Modifying the BaFL pipeline allowed us to remove noise and systematic errors, improving the power of this study, which had a small sample size. Each bioinformatics approach revealed DE genes not predicted by the other; subsequent PCR assays confirmed seven of twelve candidates, a relatively high success rate.

## Background

In sporadic Amyotrophic Lateral Sclerosis (sALS), multiple pathways are implicated to different degrees across individuals, probably due to environmental and genetic factors that are not currently understood [1,2]. We are interested in identifying strong biomarkers (insensitive to genetic background) from blood, which is routinely obtained during physical exams. Microarrays are particularly suitable when searching for common markers in polygenic diseases like sALS, and have been used to obtain both transcript and genotyping profiles. Because the platforms were the first fully parallel instruments for assessing cell state, large data sets have been created and widely shared [3-6]. Despite their ubiquity and frequent success, the correct handling of microarray measurements is still subject to debate, and conflicting interpretations are common [3]. Many factors have been identified as contributing to the inconsistent outcomes. An individual’s divergence from the ‘reference standard’ used in platform design is one factor we cannot control *a priori*[7], but biophysical properties of the sensors, or probes, are also important factors [3-5] that do not change with the sample, and cause considerable differences when comparing arrays from different suppliers. Probe placement with respect to transcripts, and which isoforms are detected, vary by supplier [6]. Noise has both biological and technical sources, from factors such as availability of a homogeneous sample and the completeness of amplification and fragmentation steps [8]. Determining how these factors affect measurements is amenable to modelling although to date no single approach has achieved dominance; however it is not disputed that removal of flawed measurements improves the detection of meaningful patterns in the data [1].

We had available microarray data previously produced in a study by Mougeot et al. [9]. Briefly, this study used a pooled reference design and compared expression levels of genes in each of 22 samples to that pool, 11 from patients with ALS and 11 from age- and gender-matched healthy controls. The samples were purified peripheral blood lymphocytes (PBLs). For some of the samples RNA remained for follow-up testing, although the amounts were very small. Because of the limited number of samples we wanted an array analysis method that removes as many sources of bias as possible. To this end we characterized the long-oligonucleotide probes used on the Agilent 4x44k human arrays for sequence and structural properties that confound measurement interpretation. We based the method on steps used in the Affymetrix-specific BaFL pipeline [10]. Compared to Affymetrix arrays, probes on Agilent arrays are longer (60-mers) and only 1–2 probes are present per gene compared to Affymetrix arrays 11–16 probes (25-mers) per gene. Accommodating these differences required modifying parameters in the BaFL algorithms used to identify and map probes to the genome. For example in SNP filtering the polymorphisms between probe and target affect binding, but the number of such mismatches that would eliminate the signal depends on the length of the duplex [9-11]. Testing for internal probe structures that compete with duplex formation required both length and hybridization condition adjustments [11-13]. Two new steps were added, one to identify homopolymeric G runs (> 3), which often lead to high signal uncorrelated with concentration [14] and another to check whether probes map to families of repeat elements. We also modified the criteria by which probes ‘correctly’ map to the reference genome: Affymetrix probes often map to untranslated regions or introns, while Agilent probes are intended to map to a single exon (eliminating sensitivity to alternate splice forms); we removed probes that violated the design constraint.

In addition to applying the BaFL method to our data we performed a parallel analysis with the TM4 [15] package, which uses statistical criteria to identify and remove poor measurements [16]. We used two of the four modules: the MIDAS (Microarray Data Analysis System) application which includes several normalization steps and low-intensity filtering, and the Significance Analysis of Microarrays (SAM) [17] package for predicting differentially expressed (DE) genes. TM4 removes measurements in an experiment-specific way based on their behaviour, while LO-BaFL removes probes in an experiment-neutral way based on their physical properties and measurements based on scanner detection limits. When we performed the analyses we did not know to what extent the DE gene lists produced by the two methods would coincide.

For rare diseases, such as sALS, it is difficult to obtain the large sample sizes needed to identify factors having moderate effects: in our study we had only 22 samples, 11 from patients with confirmed sALS and 11 from age and gender-matched healthy controls. We hoped to increase these numbers by combining our study with studies from other researchers, both to increase our statistical power and to broaden the effective population for any markers. We were unable to identify another sALS microarray study with publically available raw data. However, we did identify an Agilent 4x44k microarray expression data set from a Coronary Artery Disease (CAD) [18] study whose healthy control samples came from mixed white blood cells from a population with similar demographics to ours [19]. In brief, this study examined samples from 17 CAD patients and 14 healthy age- and gender-matched Controls based on circulating blood cells (fractionated peripheral blood mononuclear cells) collected from participants, that included qRT-PCR validation of predicted DE genes. We tested whether LO-BaFL predicted the validated set of DE genes from the CAD study to make sure the approach could mimic successful results, and to compare the our small set of healthy control data to an independently measured set to ensure that we had a good baseline for predicting DE genes in the sALS arrays.

The effectiveness of a data cleansing method can be assessed in a number of ways, including improvements in the accuracy of subsequent data mining efforts, whether detecting differential expression or sample classification [20]. The most credible confirmations are sample-based, using an independent assay (usually qRT-PCR), but concordance over multiple data sets or reports on the level of specific genes are also accepted. With small amounts of amplified sALS material remaining from the microarray study we tested the top DE predictions by qRT-PCR assays [21]. Microarray and qRT-PCR results were declared concordant when the direction and the degree of change in expression compared to a control gene were accurately captured [22]. The CAD study also used qRT-PCR to test predictions, and we accepted the accuracy of the reported results [19]. We then performed a literature search for reports on expression of specific genes, or pathway and interaction data known to be important in sALS [2,23,24].

## Results and discussion

### Probe filtering

For each LO-BaFL step the output is available along with the final results of our analyses, in the Supplementary Material section: http://webpages.uncc.edu/~cbaciu/LO-BaFL/supplementary_data.html. Summarized results are reported below, and implementation details are in the Materials and Methods.

#### The cross-hybridization filter

Scanning for near-perfect and perfect matches between the 41,000 Agilent probes and HRG36.1 (Human Reference Genome build 36.1), identified 370,139 hits, or an average of ~9 matches per probe. The full list of matches is available on the project Web site/Cleansing process/tera_blast_results/tera_blast_raw.zip. In a gene expression array the impact of a perfect match to a secondary target depends on whether it is an expressed sequence: most cross-hybridization was to unexpressed regions. Perfect matches of probes mapped to protein-coding genes, with a few exceptions (described in (iii)).

#### The duplex stability filter

Using the Kane criteria and OligoArrayAux values, ~8.63 % of the probes had cross-hybridizing partners to expressed regions that would produce mixed measurements (detectable signal coming from multiple targets); such probes were flagged for removal [25,26]. The set of allowed probes is available on the project Web site under: ‘Cleansing Process/cross-hybridization_filter/total_probes_no_crosshyb.csv’.

#### The exon-specific and target loss filters

There are 407 probes that no longer map to an exon in HRG36.1, a list is given in ‘probes_info_no_pm_no_crosshyb_not_mapped.csv’ in Supplementary Data/Cleansing Process/loss_of_target_filter; these were flagged for removal. While several of these probes do map properly to transcripts, they must cross exon junctions. They are clearly described and could be returned to the data pool if the isoform were known to be present.

#### The SNP filter

Of the remaining probes, ~2.53 % map to targets containing 4 or more SNPs, so they were removed from the list of allowed probes. The file showing the probes with major/minor alleles for each SNP position is in Supplementary Material/Cleansing process/SNP filter/snp_info_probes_gt_4snp.csv. Should samples be SNP qualified, probes can be returned to the allowed list. We include another file, ‘agilent_probe_info_3SNPs.csv’ that describes probes with one to three SNPs.

#### The OligoArrayAux filter

For 60-mers under the stated hybridization conditions, setting monomer folding stability to ΔG = −5.2 kcal mol^-1^ separates probes that are less responsive to changes in target concentration from the majority of probes that are similar in their response to increasing target concentration. Applying this filter resulted in removal of ~21.5 % of the probes, listed in the Supplementary Material file under ‘DeltaG_filter’ tables.

#### The poly-G filter

There are 4,742 poly-G containing probes in the original array design (see file ‘log_signal_4G_probes_total_no_filters.csv’, under polyG_filter of Cleansing Process in Supplementary Material). Of these ~2.3 % had unusually high intensity (log_10_(I) > 4.5) vs. 1.5 % with this intensity in the probes without this feature. At the other extreme, 16 % of poly(G) probes had log_10_(I) < 1.1 vs. 7.7 % of the probes with no poly-G stretch. Since these probes have unusual behaviour at both extremes, we examined the final probe list for the frequency of this feature: only 19 poly-G containing probes were still present. Since 8 of these probes show very high signal (log_10_(I) > 4.5) we removed all of them.

#### The repeated element filter

A screen of the probes for LINE, SINE and Alu subsequences was performed against the TranspoGene database [27]; unlike our results with the Affymetrix human expression and SNP6.0 array probes, no matches were identified (data not shown).

**Figure 1:**
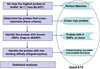
**The LO-BaFL method flowchart: the steps are given on the left; comments to the right indicate where intermediate datasets were stored in the project database.** Note that for each step the output has been made available as flat files.

A summary of the entire workflow is shown in Figure 1, and the summary of the pipeline effects (shown as percentage of total filtered out per step) is shown in Table 1.

**Table 1:** The percentage of probes removed per filtering step by LO-BaFL (on Agilent human expression 4 x 44 k microarrays) in comparison with the percentage removed by the BaFL method (on Affymetrix human expression U133Av2 arrays)

**Applied filter**	**% Probes filtered out (LO-BaFL)**	**% Probes filtered out (BaFL)**[7]
Cross-hybridization	8.63 %	60.30 %
Loss of target	0.99 %	2.19 %
SNP	2.53 %	1.78 %
ΔG	21.46 %	5.17 %

### Measurement filtering

**Figure 2:**
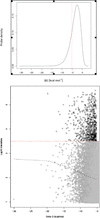
**A graphical representation of ΔG**_**cut-off **_**and of ΔG vs. Probe Signal.** Top panel shows **Δ**G_cut-off_ results: the probes having **Δ**G < −5.2 kcal mol^-1^ fall to the left of the red line, these were filtered out (21.5 %). Bottom panel shows **Δ**G vs. Probe signal: the red line denotes background cut-off value; the grey line is the Lowess smoothing line between **Δ**G and log_10_ intensities; grey dots represent the probes with very stable structures that have been eliminated in the process; black dots represent the probes with signal higher than the background cut-off value and **Δ**G < −5.2 kcal mol^-1^.

Estimating the background and then filtering for measurements that fall below it is experiment specific. To retain a probe it must have a valid measurement in every array in a sample class. In the sALS study ~27,000 probes were removed by this filter; see Supplementary Material/Cleansing Process/Instrument_cutoff/all_samples_gt_instrument_cut-off_log_intens.csv for the list. In our sALS arrays, log_10_(I)_mean_ and log_10_(I)_median_ for this set of probes yielded values of 3.51 and 3.49 respectively, while for the CAD samples the values were 3.0 and 2.9 respectively. This is ten-fold higher than the value we find for most experiments using Affymetrix scanners (Thompson, personal report) but the instruments are different. Because we lost so many genes from this filter we estimated a second cut-off value, using unexpressed genes having ΔG < −10 kcal mol^-1^ which results in a background estimate value that is about 10-fold lower (200–300 fluorescent units). In comparison, the TM4 pipeline uses Lowess smoothing to estimate background, shown in Figure 2 for the ALS samples (lower panel), and Additional file 1: Figure S1 for the CAD data (see ‘Supplementary Data/CAD study’). The Lowess approximations fall between 200–300 fluorescent units, similar to the less stringent LO-BaFL estimate above. We tested whether using the less stringent cut-off would improve the concordance in DE genes between LO-BaFL and TM4 predictions, since more similar starting gene lists might result in more similar outcomes. Since no improvement occurred (data not shown) we kept the stringent background estimate value for subsequent analyses.

### Sample Filtering

All of the samples in the sALS experiment passed outlier detection tests, including those whose residual samples had poor RNA Integrity Numbers (RIN), which we interpret to mean that the target applied to the microarray had acceptable quality, with degradation occurring during storage. CAD samples were similarly tested: no outliers were detected.

### Comparing distributions of samples and probes

**Figure 3:**
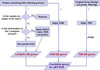
**Q-Q plot showing the distribution of the log**_**10**_**-expression values of all probes in each sample (y-axis), compared to a theoretical normal distribution (x axis), for diseased (upper) and healthy controls (lower).**

In both the CAD and sALS studies, Fisher’s test indicated unequal variances between the healthy control and diseased groups of samples. Testing measurements for the individual probes for the DE genes of interest, across replicates, in the two sample classes, using the Shapiro-Wilks test for normality [28-30], also indicated non-normality. Given the presence of unequal and non-normal distributions (see Figure 3 and Additional file 1: Figures S2, S3 in Supplementary Data/CAD Study) the non-parametric Wilcoxon two-sample test for unpaired groups [31,32] was applied.

### Predicting differentially expressed sALS genes

For input data to the Wilcoxon test we used measurements from either the 12 sALS samples with the highest quality RNA (6 in each class) or from all 22 samples (11 in each class); the latter resulted in a shorter input list as some genes were eliminated when their probe signal fell below background in a few arrays. After processing measurements with TM4 or LO-BaFL and applying the Wilcoxon test we first used the Bonferroni multiple-test correction, but were left with no significant DE genes in any of the trials. However, applying a FDR with p < 0.05 resulted in a set of significant differentially expressed genes from each method. In the output files the results for TM4 are labelled ‘TM4/W12’ and ‘TM4/W22’, results for LO-BaFL are labelled ‘LO/W12’ and ‘LO/W22’. To verify that differences were not due to differing implementations of the Wilcoxon test we also loaded LO-BaFL filtered data into SAM and selected the Wilcoxon test (results are labelled ‘SAM/W12’ and ‘SAM/W22’). The main caveat in interpreting this comparison is that there must be sufficient observations in the expected classes for each method to be valid.

The LO/W22 sALS experiment returned 87 probes as DE and the LO-W12 experiment returned a subset of 60 of those genes. For the CAD study, 386 genes were found to be differentially expressed, and this set included those confirmed by RT-PCR whose probes had not been removed by LO-BaFL filtering. The list with all DE genes for ALS is provided for each analysis in Supplementary Material/Data post filtering/DE_genes_12(or all)_samples tables. DE genes for CAD experiment are listed in Supplementary Data/CAD Study/DE_genes_CAD/DE_genes_CAD_data.csv.

Table 2 compares the six lists of 5 most significantly DE genes across the 3 analysis methods and 2 sample groupings. Clearly, the R and SAM implementations of the Wilcoxon are very similar, with SAM being more stringent since it eliminates one gene allowed by the non-permutation based algorithm. The number of samples made a large difference: only 1 of 5 genes is in common when 12 of the 22 samples were processed with LO-BaFL (that being *JUNB*) or with TM4 (the gene being *DYNLT1*). Checking the list of LO-BaFL deprecated probes shows that four of the TM4 DE genes fell below the background estimate value for LO-BaFL, explaining their absence. The final TM4-predicted gene, *DYNLT1* did not appear on the LO-BaFL list because it did not meet the p-value criterion.

**Table 2:** Comparison of genes determined to be DE in the ALS experiment by each method using either 12 or 22 samples

**List of DE genes**	**Gene/Accession**	**Description**	**p-value/SAM score**
LO/W12	FTH1/NM_002032	Ferritin, heavy polypeptide 1	1.59E-3
	JUNB/NM_002229	Jun B proto-oncogene	3.67 E-3
	B2M/NM_004048	Beta-2-microglobulin	1.54 E-3
	ACTG1/NM_001614	Poly(A) binding protein, cytoplasmic 1	3.7 E-3
	SLC25A3/NM_005888	solute carrier family 25 (mitochondrial carrier; phosphate carrier), member 3	4.46 E-3
LO/W22	EXOC3L2/NM_138568	Exocyst complex component 3 like 2	5.73 E-3
	FAU/NM_001997	Finkel-Biskis-Reilly murine sarcoma virus	1.96 E-3
	GLTSCR1/AF182077	Glioma tumor suppressor candidate region gene 1	2.56 E-3
	JUNB/NM_002229	Jun B proto-oncogene	1.24 E-3
	IRS2/NM_003749	Insulin receptor substrate 2	1.66 E-3
TM4/W12	CSE1L/NM_001316	CSE1 chromosome segregation 1-like (yeast)	3.95E-3
	NUP88/NM_002532	Nucleoporin 88 kDa	3.95E-3
	PARP1/		
	NM_001618	poly (ADP-ribose) polymerase 1	3.95E-3
	DYNC1I2/NM_001378	Dynein, cytoplasmic 1, intermediate chain 2	6.17E-3
	DYNLT1/NM_006519	Dynein, light chain, Tctex-type 1	6.48E-3
TM4/W22	IRS2/NM_003749	Insulin receptor substrate 2	1.22E-04
	SKIV2L2/NM_015360	Superkiller viralicidic activity 2-like 2 (S. cerevisiae)	1.22E-04
	DYNLT1/NM_006519	Dynein, light chain, Tctex-type 1	1.60E-04
	C12orf35/NM_018169	Chromosome 12 open reading frame 35	2.07E-04
	TARDBP/NM_007375	TAR DNA binding protein	2.68E-04
SAM/W12	FTH1/NM_002032	Ferritin, heavy polypeptide 1	1.628
	JUNB/NM_002229	Jun B proto-oncogene	1.429
	B2M/NM_004048	Beta-2-microglobulin	1.452
	ACTG1/NM_001614	Poly(A) binding protein, cytoplasmic 1	1.234
	SLC25A3/NM_005888	solute carrier family 25 (mitochondrial carrier; phosphate carrier), member 3	1.057
SAM/W22	IRS2/NM_003749	Insulin receptor substrate 2	1.182
	GLTSCR1/AF182077	Glioma tumor suppressor candidate region gene 1	1.165
	FAU/NM_001997	Finkel-Biskis-Reilly murine sarcoma virus	1.165
	EXOC3L2/NM_138568	Exocyst complex component 3-like 2	1.099
	JUNB/NM_002229	Jun B proto-oncogene	1.034

Genes were selected based on FDR significance (p < 0.05). Methods include: LO-BaFL-Wilcoxon (LO/W12, LO/W22), TM4-SAM-Wilcoxon (TM4/W12, TM4/W22) and LO-BaFL-SAM-Wilcoxon (SAM/W12, SAM/W22) as described in the text.

### DE validation by qRT-PCR

Levels of the four reference genes were stable and at the relative expression levels with respect to each other remained unchanged across all samples (data not shown). The summarized results for the DE genes are shown in Table 3. Highly elevated expression ratios are observed for *ACTG1, SKIV2L2, C12 orf35, B2M* and *DYNLT1* and more modest values for *ILKAP* and *TARDBP*. Our experimental results are in good agreement with the recent report by Mougeot et al. [9] showing by computational methods that *SKIV2L2, C12orf35, DYNLT1* are differentially expressed in PBL samples from patients with ALS vs. normal healthy control subjects. As in previous studies, *TARDBP* is among the genes with differential expression for ALS [33,34], although the contribution of the ILKAP gene led to a smaller increase in TARDBP than was predicted by TM4. Furthermore the present work reveals three novel DE genes: *ACTG1, B2M,* and *ILKAP*.

**Table 3:** The expression ratio of the genes tested in the qRT-PCR assays, determined by the Pfaffl method

**Gene Symbol**	**Gene Accession**	**Expression Ratio**
*ACTG1*	NM_001614	48.5
*SKIV2L2*	NM_015360.4	37.3
*C12orf35*	NM_018169.3	22.4
*B2M*	NM_004048	18.2
*DYNLT1*	NM_006519.1	17.4
*ILKAP*	NM_030768.2	8.8
*TARDBP*	NM_007375.3	5.6

Each analysis method predicted DE of genes excluded by the other, and in each case some of the predictions were verified with the independent assay. LO-BaFL is likely to exclude some genes that are DE because of its high background estimation value and because it removes probes that are only problematic in some populations, leading to false negatives. The example of *TARDBP* and its cross-hybridizing gene, *ILKAP*, highlights why using only TM4 is likely to lead to errors as well. In fact using both pipelines and then confirming the predictions with qRT-PCR is the best approach to achieve a complete and accurate assessment of potential biomarkers. Because LO-BAFL explicitly lists excluded probes by relevant filter, it is possible to add back particular probes when additional information warrants, a procedure not possible with TM4.

### Baseline validation by meta-analysis

**Figure 4:**
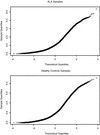
Correlation between genes in the healthy controls in ALS (y-axis) and CAD (x-axis) studies, showing the Lowess smoothing line in red.

The gene-to-gene correlation for healthy normal controls across the sALS and CAD experiments was quite high (r^2^ = 0.81), indicated visually by the flatness of the Lowess smoothing line in red (Figure 4). In both experiments the genes that are most highly expressed (e.g. *RPS2, RPLP1, RPS28, HLA-C*) and those at the lower end of detection (e.g. *CD28, CDV3, CD79A, CCD12*) are the same and characteristic of white blood cells. We carried out this analysis because determining differential expression is entirely a function of the baseline data set. In the sALS study both the diseased and reference populations were very small, and we knew that for some of the samples the quality of the remaining RNA was not high. We wanted an independent assessment of the healthy control samples: although the cell types in the sALS and CAD studies are not identical, this was the closest match we could identify. We are aware that subpopulations of cells such as PBMCs and PBLs have been shown to have distinct response signatures [35-37] so we expected to see broadly the same response with some differences and this was indeed the result. Given the general concordance of the results, we have more confidence in the results of the differential expression analysis based on this small group of sALS-normal control arrays.

### Identifying DE genes in the CAD study

For the CAD study, the list of most significant DE genes determined by LO-BaFL was compared with results reported in [19]. Two of the genes appear on both lists (*CSPG2, ALOX5*), four are close variants of the DE genes, while the remainder of the genes reported as DE in the paper were eliminated from our list based on the criteria already discussed (See ‘Supplementary Material/CAD study/comparison_with_DE_genes_CAD/comparison_LO-BaFL_CAD_DE_genes.csv’). The file listing DE genes for this experiment as determined by our method is found in the file ‘LO-BaFL_DE_genes_CAD_data.csv’, located in the directory mentioned above.

## Conclusions

We showed that results obtained from a modified probe-filtering method (LO-BaFL) gave comparable performance to a statistical filtering method (TM4), using data obtained from independent experiments that were performed using similar cell types and the same platform with similar healthy control groups, but differing disease states. For the sALS experiment the top predicted DE genes from each method, and two that cross-hybridized with one of the candidates, were tested with qRT-PCR. We confirmed the microarray DE predictions for 7 of the genes: *ACTG1, SKIV2L2, C12orf35, B2M, DYNLT1, TARDBP,* and *ILKAP*, all of which were elevated in sALS versus healthy Controls. The four control (non-DE) genes showed their predicted responses as well. These results confirm DE genes obtained in other studies [25,38,39], and adds to list of candidate biomarkers the genes *ACTG1, B2M, ILKAP*.

Employing different methods to obtain DE genes on same data set has the advantage of accounting for errors inherent to each method. The LO-BaFL pipeline identifies and eliminates cross-hybridizing probes that TM4 does not, as illustrated by *TARDBP*. On the Agilent array this probe cross-hybridizes with *IlKAP* and two other genes. TM4 assigns the signal to *TARDBP*, and here identifies it as DE, but *ILKAP* is missing entirely and we show here that it does contribute to the signal and is also differentially expressed. LO-BaFL had eliminated the probe because of cross-hybridization but by combining the results of both methods we could test all candidates with qRT-PCR. Thus we produced better DE data for *TARDBP* and new data for *ILKAP*.

Comparing normal samples from the CAD experiment [19] with those in the sALS experiment showed highly correlated expression levels in LO-BaFL filtered genes, increasing our confidence in the reliability of the baseline. In addition, the DE genes confirmed by qRT-PCR in the CAD study [19] coincided with DE genes that appeared after LO_BaFL analysis, except where probes had been eliminated by filtering. Since sALS is sporadic, and very rare, methods that allow meaningful data integration across multiple small experiments will be important to improving the power of individual studies [10,40].

## Methods

### Hardware and software

For data storage, data organization, and recording the order and parameters used in the pipeline transformations, we have used DataFATE (Data - Feature Analysis Transformation Extraction), a software system based on a relational model that includes a toolset with data import and organization tools for relational database management systems (RDBMS), tools for factor (quantitation type, QT) definition, QT set construction, and storage of data from processing steps. The RDBMS is currently PostgreSQL 8.0.3. [41]. The project instance of DataFATE was installed into a 64 bit, 22-processor, 120 GB of RAM computer running Ubuntu 9.04 version for Kernel LINUX™ 2.6.28, as the operating system. Querying, extraction and manipulation of data stored in DataFATE was carried out with scripts written with Python 2.6 [42], SQL (via PGAdminIII) [43] and R [44]. Additional software installed on this hardware and used for this project includes the TM4 microarray software suite [15], OligoArrayAux [6] for biophysical modelling. For the results using the packages TM4 and Significance Analysis of Microarrays (SAM) [17], we set up a relational database to maintain stable output of intermediate and final results of both pipelines.

### Data selection and acquisition

Microarray image files and corresponding spot intensity values for the sALS study were provided by JLM and BRB. The microarray experiment used Agilent 4x44K human genome microarrays [45] in a pooled healthy control reference design [38]. Sample and microarray processing were performed at Cogenics^TM^[39], producing arrays contrasting each sample (healthy and diseased) to the reference. The raw data sent back by Cogenics includes extracted spot intensities and the background-subtracted intensity ratios for each contrast.

A major shortcoming of many analysis methods is that they are over-tuned to a particular experiment, so that parameters that yield excellent results in one study give poor results in another. If the LO-BaFL filters are experiment neutral (except for the estimation of background) then LO-BaFL should predict the behaviour of genes from similar samples but different experiments relatively well. We identified one experiment that used human peripheral white blood cells and the same type of Agilent array. Some of the DE genes had been followed up with qRT-PCR assays, so these became our benchmark for determining whether LO-BaFL could produce a comparable DE gene list. Although the disease conditions differed, the healthy Control samples were similar and could provide a check on the quality of the sALS Control samples. The experiment studied coronary artery disease (CAD) from healthy controls (n = 14) and patients with disease (n = 27) [19]. The data is accessible at GEO, Accession No.GSE10195. To compare the behaviour of the two control groups we randomly selected six Control samples (from the original 14) and the six highest-quality Control samples from the sALS study. An anomaly in the CAD study was a number of spots with ‘negative’ intensities (often saturated spots that the software does not know how to handle), which were removed. Prior to probe filtering, all of the samples have acceptable measurements for 24,336 genes. LO-BaFL probe filtering and comparisons are described below.

### Computational methods

#### The LO-BaFL method

The steps in the pipeline described below are summarized in Figure 1.

A. In this section the probe-sequence based filters are described.

Note that the order of operations is independent for the above filters; some probes fall into multiple categories so summing over the ‘bad’ probes identified per step will give a larger number than the total number of probes removed.

Rather than remove flawed measurements from individual files, flawed probes and probes for which there were not consistent above-background measurement in all samples (discussed next) were removed from the array layout file with Aroma, ensuring consistent removal of the related measurements across all files [52].

(i) Re-map the Agilent probes to build 36.1 of the human genome using the accelerated Tera-BLAST algorithm, as implemented on a TimeLogic-Decypher [46] server. The corresponding matches were deposited into an instance of the DataFATE database. Parameters were: nucleic match = 1; nucleic mismatch = −3; open penalty = −5; extend penalty = −2; threshold significance = 10. The input and output files can be found in Supplementary Material section, at: http://webpages.uncc.edu/~cbaciu/LO-BaFL/supplementary_data.html under Input Files/agilent_fasta or Cleansing Process/tera_blast_results.

(ii) Determine the cross-hybridization potential of probes to other sites in the genome, using the Kane criteria [47]. Briefly this is an empirical rule stating that any target sequence with similarity greater than 75 % across the length of a probe can contribute a detectable amount of signal to the total intensity. This rule includes some constraints concerning the positions and lengths of mismatch regions. For a probe to cross-hybridize, we input the following conditions: percent identity ≥ 85 %; presence of 50 matches out of 60 possible; minimum of 15 consecutive nucleotides in the Agilent probe sequence. We stored the output, consisting of all the Kane-criteria cross-hybridizing probes into DataFATE

(iii) Identify probes that no longer anchor to the reference genome. This information is acquired when a TeraProbe query returns ‘no hit’, and this is stored as an explicit type. We note that our criteria include restricting location to one exon.

(iv) Identify SNPs and short indels known to occur in the probe-binding region. Probes were mapped to the human instance of dbSNP [48], taking all possible alternate alleles into consideration. The minimum number of SNPs expected to significantly degrade the signal is a parameter in the BaFL pipeline. Using the Kane criteria, the presence of six SNPs will reduce the signal to the point of background, but the presence of any SNP will cause the signal to reflect both sequence variation and transcript concentration and the question of degree is not simple since it depends on sequence context and competition. For the case study we set the ‘deprecate’ flag to 4 SNPs or more, assuming that this many competing alleles would make the intensity information useless for expression analysis. All of the information was retained, however, so another researcher could modify the number of acceptable SNPs and reintroduce probes.

(v) Employ the OligoArrayAux software [6] to determine the free energy of internal probe structures (monomers) versus heterodimers. Parameters chosen were: temperature 55 to 62°C, concentrations of 1.0 M Na^+^ and 0.0 M Mg^++.^ Output was used to define probes that are less responsive to target (high concentrations are required to compete with monomer structure) under experimental conditions. Heterodimers with low stability under specific experimental conditions also do not yield signal [49-51]. The predicted value of the most stable form is stored in the database as an attribute of the probe, allowing adjustment of the cut-off value.

(vi) Identify G-runs. It has been shown for Affymetrix arrays that four or more consecutive guanines (G-runs) may lead to unusual probe structures that cause very high signal [14]. We identified the probes with this feature.

(vii) The presence of any member of the transposable elements family, short (SINE), long (LINE) or primate-specific (Alu) repeat elements, can have a great effect on gene expression. Using the TranspoGene database [28] we examined the entire set of genes for these elements; none were identified. Although this seems redundant with the cross-hybridization check, for Affymetrix SNP6.0 probes we found that the BLAST output against the full genome was incomplete and a number of matches were identified using the TranspoGene database as target.

B. Background (Noise Estimation)

We were unable to find technical specifications for the linear detection range of the Agilent scanner so we had to estimate the value [52]. This is done by first selecting probes with very stable monomer structure, since they are less responsive to increasing target concentration, and then filtering this group for genes that are not expected to be expressed in healthy control samples. The mean intensity of measurements of these probes in each sample class is used to estimate the background (given in Supplementary Data/Cleansing process/Determining_instrument_cutoff/delta_g_mean_and_log_int_probes_no_crosshyb.csv). Once this value has been determined, measurements are filtered based on whether they exceed that value. Candidate probes were determined based on queries for uniqueness and free energy; genes not expected to be expressed were identified by inspecting the CAD results. Measurement values were then retrieved, from each dataset independently, and the mean, median and Lowess values were determined.

C. Sample Outlier Detection

The sALS experiment used a pooled reference design, so we determined the reproducibility of the reference signal across all of the samples. An outlier was defined as any sample whose signal fell outside two standard deviations of the class mean for either the average signal per probe or the average number of probes per array. We compared the signal intensities for both the normal and diseased signal to the mean and variance of each class. Two contrasts were examined:

(i) Sample to class comparison: Using only accepted probes with measurements above the threshold in a sample class, determine the mean signal per probe per array and across all arrays in the class. Samples whose probe-signal mean falls more than two standard deviations outside of the array mean are rejected. Determine the number of acceptable probes yielding good measurements per array and across all arrays in the class, and similarly reject any sample whose probe-number mean falls more than two standard deviations from the class mean.

(ii) Sample distribution comparison: Determine whether the remaining values fall in a normal distribution, and compare the within- category and between-category distributions. If not normal this would suggest that a log transformation might be advisable (but our values for the sALS experiment were already log_10_ transformed).

### Statistical analyses

**Figure 5:**
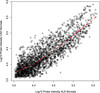
The computational experiment workflow, showing decision points for the algorithmic steps making up the method used, ending in the determination of differentially expressed genes (LO-BaFL vs. TM4 for processing and R-Wilcoxon vs. Sam-Wilcoxon for test implementation).

The sequences of steps performed as statistical analyses are shown in Figure 5.

A. Measurement distribution and normality

We used QQ plots to examine the distribution of probes over samples and sample classes. There are two common ways to employ QQ plots on microarrays. Yang and Speed [53] proposed testing measurements from all probes on an array-by-array basis, assuming that where there were large differences the samples should not be compared. Wit and McClure [54] point out that it is the distribution of signal across samples for a single gene that should be tested, assuming enough replicates exist and that the behaviour of the complete set of measurements is irrelevant to the specific target being tested, assuming enough replicates exist. For the probes that passed the LO-BaFL method, the measurements were tested for distribution. We note that individual normal and diseased samples were labelled with Cy3 and the pooled reference was labelled with Cy5, which means the pooled reference group had twice as many members, which was taken into account. The Shapiro-Wilks test [28-30] implemented in R was used to check for normal distribution within and between sample classes. Since the results of both experiments show a non-normal distribution (data not shown), the Wilcoxon non-parametric test [31,32] for unpaired groups was applied in the following step.

B. Detection and Significance of Differential Expression

Microarrays confront the analyst with the problem of testing multiple hypotheses within the same data matrix. We addressed this issue using the Benjamini and Hochberg FDR procedure [55], as implemented in R. The output consists of a list of DE genes and associated p-values. The R scripts for statistical analysis and the output file with DE genes can be found in Supplementary Material/Scripts/stats_R.txt. The inputs varied slightly between the two processing packages, as described below.

TM4 modifies the measurements to normalize and standardize the signal in a sample class (total intensity normalization, Lowess normalization, standard deviation regularization, filtering for the lowest 5 % intensity signals, and signal detection boundaries of 100 for Cy5 and Cy3 intensities); these values are used by the Significance Analysis of Microarray (SAM) package using the Wilcoxon non-parametric test with permutation (set to 100) [31,32] to identify the differentially expressed genes. The output is a list of DE genes and associated p-values (results are labelled ‘TM4/W12’ and ‘TM4/W22’ respectively).

The LO-BaFL workflow simply used the log_10_ transformed measurements of accepted probes since the arrays were found to have similar background and total intensity. These values were submitted to the Wilcoxon non-parametric *t*-test implemented in R (which does not use permutation) to determine DE genes for both the high RIN and full set of samples (result sets are ‘LO/W12’ and ‘LO/W22’ respectively).

### Wet-lab methods

#### Gene selection

In the absence of a calibration standard the actual expression levels of genes in the individual samples are not readily available. Thus the wet-lab work had two goals: determine the level of expression that a microarray value yields in a qRT-PCR assay; determine whether either analysis method was accurate in its predictions of the difference in expression levels between genes in normal and diseased samples. Table 3 shows the genes selected and their status as reference gene or candidate DE gene. Those marked as ‘reference’ are expected to be expressed in PBLs at moderate levels (varying somewhat between genes) and consistent levels across all samples.

### Quantitative and qualitative assessment of RNA

From 2007 through March 2008, the blood samples used for microarray and PCR analysis were collected at Carolinas Neuromuscular/ALS-MDA Center with approval by the IRB at Carolinas Medical Center. Informed consent was obtained from all participants in this study. Detailed information about patient status and early stages of sample processing are provided in [9]. The isolated RNA from peripheral blood lymphocytes samples of healthy controls and ALS patients, stored at -80 °C, provided by the ALS Biomarker Laboratory, Carolinas Neuromuscular/ALS-MDA Center, Department of Neurology, Carolinas Medical Center (J-LM, BRB) was qualitatively checked using the Agilent 2100 Bioanalyzer (Agilent Technologies, Santa Clara, CA), and quantified with the Nanodrop ND-1000 (ThermoFisher Scientific, Waltham, MA). We carried forward only those samples that satisfied the condition that RIN >5.0.

#### cDNA synthesis and QC

A properly designed qRT-PCR assay includes calibration genes for the sample and assay [56]. For our references we selected *UBE2Z*, *PGK1*, *COX4I1*, *SRRM1*. A reference is context specific: these were selected based on an apparently consistent level of expression in the microarray data across sample classes and individuals, over a moderate range of concentrations. For all genes the PCR primers were designed to bridge the position of the array probe where possible (the set cross-hybridizing with the TARDBP probe had to be adjusted) and tested against genomic DNA. The list of test qRT-PCR genes and the assay primers is given in Table 4**.**

**Table 4:** Genes used in the qRT-PCR assays, and the sequence of the PCR primers used in the assays

**Gene information**	**Gene role**	**Forward primer (5` to 3`)Reverse primer (3` to 5`)**
*UBE2Z*, NM_023079.3	Reference gene	GCAGAGCATGTCTGGCATAG TTCTCCTTCTGCCAAAACAAA
*PGK1*, NM_000291.3	Reference gene	TGCATCTCCACTTGGCATTA TGGGATCTTGAAGAATGTATGC
*SRRM1*, NM_005839.3	Reference gene	GGAAATCCTTGGGTTTGAAGA GGCCACAGTTCTCCCATAAA
*COX4I1*, NM_001861.2	Reference gene	GGCACTGAAGGAGAAGGAGA GGGCCGTACACATAGTGCTT
*B2M*, NM_004048	DE gene determined by LO-BaFL/SAM	GATGAGTATGCCTGCCGTGTG CAATCCAAATGCGGCATCT
*ACTG1*, NM_001614	DE gene determined by LO-BaFL/SAM	AGAGGCTGGCAAGAACCAGTTGTT CAATGACGTGTTGCTGGGGCCT
*DYNLT1*, NM_006519.1	DE gene determined by TM4 analysis	CCAGCCTATGGCCTTTCTCCTTTTGT CAACGCAGGCTGCAGGTGAC
*SKIV2L2*, NM_015360.4	DE gene determined by TM4 analysis	TGCAGAAGGAATCACCAAAA ATGGGAGAACCAAATCCACA
*C12orf35,* NM_018169.3	DE gene determined by TM4 analysis	CGGGGAAACAAGGTATTTGA TTCACATCACAGTGGGCATT
*TARDBP*, NM_007375.3	DE gene determined by TM4 analysis	TTTGCTGCAGTTCTGTGTCC TCCATCTCAAAAGGTCAAAA
*ILKAP*, NM_030768.2	Cross-hybridizing gene with TARDBP	CACAGGAGTACACAAAACACAC TGCGGATAGGGCACTGAG

To test primers and conditions for the qRT-PCR assays, we extracted RNA from an anonymous sample of white blood cells, suspended in Triazol and kept at -80 °C, using the AllPrep DNA/RNA Mini Kit from Qiagen (QIAGEN, Valencia, CA), following the manufacturer’s instructions. This RNA was qualified and quantified as above. We then synthesized double-stranded cDNA from the ALS samples that passed the RNA quality/quantity test (6 normal controls and 6 diseased) and from the control RNA, using the Full Spectrum^TM^ Complete Transcriptome RNA Amplification Kit from System Biosciences (System Biosciences, Mountain View, CA), according to the manual. After quantification of the yield, and standardization of the concentrations, the cDNA products were qualified by determining whether the four reference gene PCR primers yielded the expected size product on 12 % Acrylamide (native) gels [57]. Even where the starting concentration of RNA was low, e.g., 20 ng, we obtained good yields of cDNA and strong signal from the reference genes. The PCR reaction conditions were as follows, per 50ul final volume: 5.0 μl/reaction of 10X Buffer, 3.5 μl/reaction MgCl_2_, 50 mM stock solution, for Mg^++^ 3.5 mM final concentration), 2.5 μl/reaction dNTP mixture (all reaction components from Invitrogen^TM^ Life Technologies, Grand Island, NY, 10 mM stock, 2.5 mM final), 0.5 μl/reaction DNA Taq Polymerase (New England BioLabs® Inc., Ipswich, MA) 5U/mL in stock; final concentration of 2U/mL), 2.5 μl/reaction forward and reversed primers (Eurofins mwg|operon, Huntsville, AL) solubilised with DNA Suspension Buffer for a concentration of 100 mM stock; final concentration of 5 mM), 2.0 μl/reaction cDNA as template (100 ng). The GeneAmp® PCR System 9700 from Applied Biosystems (ABI, Life Technologies, Grand Island NY) was set up to the following profile: the initial DNA denaturation at 95 °C for 5 min; 30 cycles of denaturation at 94 °C for 30 s, primer annealing at 57 °C for 30 s and extension at 72 °C for 30 s; a final elongation at 72 °C for 4 min and a 4 °C hold.

### Primer design and synthesis

The primers were designed using Primer3 software ( http://frodo.wi.mit.edu/primer3/) in combination with NCBI Primer-BLAST ( http://www.ncbi.nlm.nih.gov/tools/primer-blast/) to check for specificity. Whenever possible, the primers were designed to bridge the positions occupied by the corresponding Agilent probes, in order to account for sensitivity to transcriptional isoforms. Primers were purchased in dry form from Eurofins mwg|operon and resuspended in DNA Suspension Buffer; concentrations were verified with the Nanodrop1000 spectrophotometer, length and purity were gel-verified using 12 % Acrylamide in 1X TBE buffer [57]. PCR performance was checked with the cDNA made from the control RNA. PCR conditions were optimized by changing the Mg^++^ concentration in a range of 2.5 – 4.0 μM, the annealing temperature in the interval 55 °C - 60 °C and varying the dNTP mixture concentration from 2.5 μM - 3.5 μM. Where necessary new primers were designed and run again through the QC protocol mentioned above.

### Real Time qRT-PCR

Before proceeding with qRT-PCR assays with sALS samples, we verified the quality of the reference standards for each gene product and optimized the PCR reaction conditions. By adjusting the primer annealing temperature, the concentration of Mg^++^ and the dNTP mixture concentration, we obtained products that could be amplified well using a common set of PCR reaction and cycling conditions. These are: annealing temperature = 57 °C; Mg^++^ concentration = 3.5 μM; dNTP mixture concentration = 2.5 μM; primer mixture concentration = 5.0 μM.

Assays were set up in parallel, taking PCR reagents from a master mix to amplify the gene product reference at known concentrations against a mass-titration of a sample’s cDNA product [21]. We used the following reagents: 10.0 μl/well of iQ^TM^ SYBR® Green Supermix from Bio-Rad (Bio-Rad Life Science, Hercules, CA) that includes 2X reaction buffer with dNTPs, iTaq DNA Polymerase, 6 mM MgCl_2_, SYBR Green I, fluorescein and stabilizers according to the Bio-Rad specifications; forward and reversed primer mixture (2.0 μl/well at 5 mM); 5.0 μl/well of template solution which variously contained the standard gene, the unknowns or Accugene water (Lonza AG, Allendale, NJ), and 3.0 μl/well Accugene water in final reaction volumes of 20.0 μl/well. Titration series were set up as follows: six 10- fold serial dilutions of the gene product reference and of the samples, in triplicates, with negative controls in all series to identify any cross-contamination problems. The reactions were set up in 96-well clear Multiplate® PCR Plates covered with iCycler iQ^TM^ Optical Tape (Bio-Rad Life Sciences, Hercules, CA). The instrument employed for these reactions was MyiQ Single-Color Real-Time PCR Detection System from Bio-Rad. We used a 2-step protocol with the following profile: Cycle 1:(1X) Step 1: 95.0 °C for 03:00; Cycle 2:(40X) Step 1: 94.0 °C for 00:15; Step 2: 57.0 °C for 00:30; data collection and real-time analysis enabled; Step 3: 72.0 °C for 00:15; Cycle 3: (1X) Step 1: 95.0 °C for 01:00; Cycle 4:(1X) Step 1: 55.0 °C for 01:00. Data were analysed according to the method of Pfaffl [58].

## Competing interests

The authors declare no conflict of interest.

## Authors' contributions

JWW designed the experiments; CB performed the research; J-L.M and BRB provided the PBLs samples, the raw ALS microarray data and contributed to TM4 data analysis; KJT helped with data analysis and figures; CB and JWW wrote the paper. All authors read and approved the final manuscript.

## Supplementary Material

Additional file 1 **Supplementary Material can be freely accessed at the author’s project Website:http://webpages.uncc.edu/~cbaciu/LO-BaFL/supplementary_data.html.** Click here for file
