# Supplementary figures and images for: The LO-BaFL method and ALS microarray expression analysis
Source: BMC Bioinformatics. 2012 Sep 24;13:244. doi: 10.1186/1471-2105-13-244 (PMC3526454; doi:10.1186/1471-2105-13-244)

**Normal Distribution of Diseased Samples in CAD Study,After Filters**

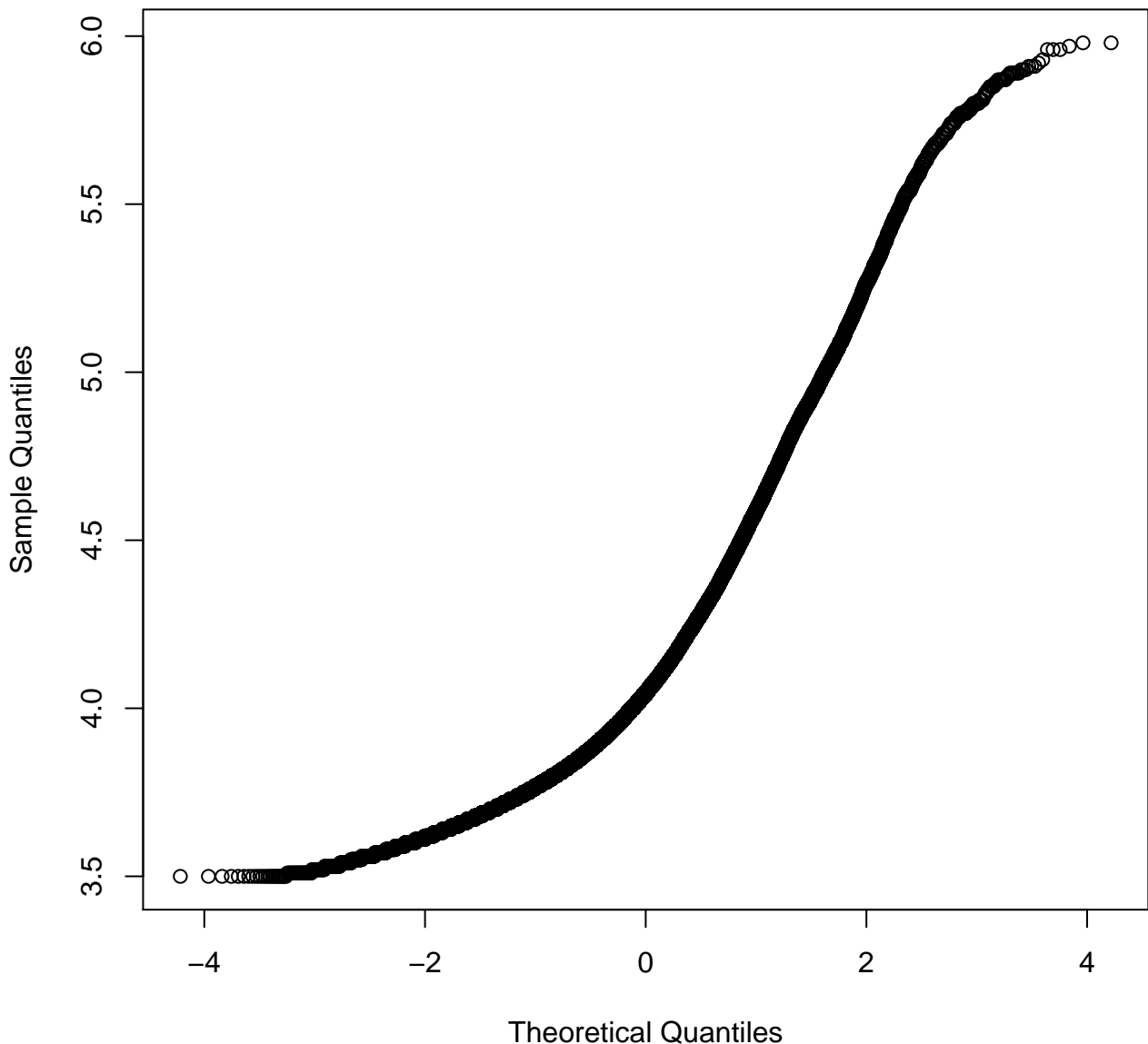

Supplement: Additional file 1 — Supplementary Material can be freely accessed at the author’s project Website:http://webpages.uncc.edu/~cbaciu/LO-BaFL/supplementary_data.html. [file 1471-2105-13-244-S1.pdf]
